# Mapping inspiration in human resources:  A systematic review of themes and approaches

**DOI:** 10.12688/f1000research.128853.1

**Published:** 2023-06-16

**Authors:** Vagisha, James P S, Vishwanathan Iyer

**Affiliations:** 1 Manipal Academy of Higher Education, Manipal, Karnataka, 576104, India; 2 T A Pai Management Institute, Manipal, Karnataka, 576104, India; 3 Jagdish Sheth School of Management, Bengaluru, Karnataka, 560100, India; 4 Great Lakes Institute of Management, Chennai, 603102, India

**Keywords:** inspiration, human resource management, systematic literature review

## Abstract

**Background:** This study was motivated by the need to invigorate research on inspiration, especially within the domain of management. The authors’ objective was to devise a unifying structure for theory building and provide an overview of emergent constructs on inspiration research. Thus, the incremental contribution of the study is that the authors reviewed extant relevant literature and enhanced the focus on research on inspiration in management.

**Methods:**  We performed a literature search on empirical studies on inspiration from 15 June to 31 August 2022. We retrieved English articles published between 2003 and 2022. The information sources were Ebscohost, ProQuest, Science Direct, and Scopus.  Risk of bias was assessed regarding review methods and the relevance of review to the research questions. We developed a data extraction sheet for the data collection process, considering the systematic review goals to ensure that all the pertinent data was retrieved.

**Results:** Six out of 224 articles were identified for the final review. The excluded articles did not meet the either one or all of the inclusion criteria. The results revealed that there is a vast knowledge gap awaiting empirical research which can have a far-reaching impact on society and management; for instance, the impact of inspiration on performance and the role of moderators such as spirituality, visioning capability, gender variation, and linguistic proficiency.

**Conclusion:**  This study recommends that research on inspiration focuses to crystallize inspiration as a construct, identify various dimensions of inspiration, and then firm up a general theory of inspiration with robust empirical evidence. There is a need to expand the scope of the IS by developing and trying out newer competing scales.

## Introduction

Inspiration plays a vital role in the life of people and organizations. In the organizational context, inspiration is the responsibility of leadership. The role of leaders to motivate and inspire people is often considered the key to productivity ( Conger, 1991). The era of managing by inspiration is gradually replacing the era of managing by control ( Conger, 1991). Inspirational leadership ( Molenberghs, 2015) is considered the appropriate leadership style when workers are worried, production is tenuous, and business is unstable. Therefore, inspirational leadership is needed most during economic downturns, uncertainties in world affairs, or in case of unexpected natural disasters (e.g., a tornado or an earthquake) or acts of terrorism (e.g., 9/11) ( Angelo, 2020).

The role of inspiration is even more crucial when organizations must embark upon change and handle ambiguity in times such as COVID-19 ( Joly, 2020) which underscored the importance of inspiring people to ensure high productivity ( Tabrizi, 2020) and has tested leaders on all fronts on how they lead their employees ( Joly, 2020; Goffee and Jones, 2000). Despite the hardships and risks, millions of frontline workers continued to do their jobs well in sub-optimal conditions, and COVID-like situations, Monkeypox, or wars/threats of war continue to loom large not to speak of contemporary challenges such as quiet quitting. These call for the best practices ( Tomer and Kane, 2020), and inspiration could be an important influencer of such practices.

A detailed search on inspiration from various sites such as (Ebsco, Proquest, and Science Direct), suggests that the concept of inspiration has been theorized sparsely. However, the term is referred to frequently in the domains of religion, creativity, interpersonal relationship, and leadership and often as a loose term to describe things that evoke something special in us; for example, we use terms such as inspired by a painting, a person, a waterfall, a writing, celestial beauty, etc. The initial discourses of inspiration find their place in theology as an extension of revelation. The term ‘revelation’ refers to the declaration of divine knowledge to the individual, whereas inspiration refers to transmitting the revelation into written form with invariable truths ( Aquinas, 1950). This theological account of inspiration is a typical precursor of the tripartite conceptualization of inspiration ( Thrash and Elliot, 2003) which consists of transcendence, evocation, and motivation. Inspiration implies motivation, which is to say that inspiration involves the energization and direction of behaviour ( Elliot, 1997); however, inspiration is evoked, rather than initiated directly through an act of will. Though the trigger of inspiration is often invisible, perhaps because research has not unravelled it, the result of inspiration involves a transcendence of the ordinary preoccupations or limitations of human agency ( Thrash and Elliot, 2003).

The word inspiration has its roots in biology where the term means drawing of breath and is metaphorically equivalent to energizing ( Thrash and Elliot, 2003). A semblance of use of the term in academic literature may be found in the work of Bakker which refers to vigour, which is one of the dimensions of employee engagement, other two being dedication and absorption Bakker (2008). While vigour implies energy for action, absorption, is considered to lead to enduring motivation and focus, and energization for a course of behaviour ( Thrash and Elliot, 2003; Elliot, 1997). Prior to the work of Thrash and Elliot (2003), Bass had coined the term ‘inspirational motivation’ in the leadership context ( Bass, 1999). Inspirational motivation is one of the components of transformational leadership, others being idealized influence, intellectual stimulation, and individualized consideration ( Bass, 1999). Inspirational motivation stands out as a unique component of transformational leadership, suggesting that the role of a leader goes beyond the well-researched concept of motivation. Literature around transformational leadership suggests that such leaders can beat resistance by inspiring people ( Edmondson, 2002; Faupel and Süß, 2019; Lam, 2002; Joshi *et al.*, 2009). Bass (1988) further suggested that an inspirational leader “employs persuasive appeals and arouses emotional acceptance” (p. 22). Such inspirational appeal aims to “generate enthusiasm and develop commitment” ( Yukl, 1999), which is a major role in leadership. Although Bass (1998, 1999), Yukl (1999), Cho and Dansereau (2010) and several other authors have contributed to inspiration through the prism of transformational leadership ( Gilley and Maycunich, 2000; Popper and Lipshitz, 2000; Tichy and DeVanna, 1990), none of them have explained what inspiration is. Rather, they preferred to combine the word ‘motivation’ with ‘inspiration’, perhaps because motivation was a well-established construct by the time Bass and others embarked upon research on transformational leadership, whereas inspiration was not.

Inspirational motivation leads to extra effort beyond what motivation can achieve ( Densten, 2002). Inspirational motivation uses vision of the leader ( Bass and Avolio, 1990) and helps organizations need to seize opportunities for growth and development ( Conger, 1991). Inspirational motivation has been empirically linked not only to extra effort as discussed above, but also to ethical behaviour ( Bass and Steidlmeier, 1999), learning orientation, and project success ( Banerji and Krishnan, 2000; Coad and Berry, 1998; Thite, 1999). However, extra effort is the true differentiation of inspirational motivation and is central to the discriminant validity of inspirational motivation ( Densten, 2002). This is because the extra effort leads to the ‘augmentation effect’ of inspirational motivation ( Waldman *et al.*, 1990). This augmentation effect accounts for the unique variance over and above what is accounted for by transactional leadership. Studies that show a high correlation between inspirational motivation and extra effort also exist ( Bass and Avolio, 1990; Hater and Bass, 1988; Howell and Avolio, 1993). Despite the importance of inspirational motivation, the construct validity of inspirational motivation has been questioned ( Densten, 2002). The basic question has been whether inspirational motivation is uni-dimensional ( Hinkin and Tracey, 1999) as inspirational motivation has been found to be loading on three items including motivating elements and future-oriented theme that is based on open, verbal communication ( Grant and Hofmann, 2011) in factor analysis ( Hinkin and Tracey, 1999).

One approach to address the uni-dimensionality issue has been to explore inspirational motivation as image-based concept. This has been attempted by Lord and Emrich (2000) using ‘Martindale’s Regressive Imagery Dictionary’ ( Martindale, 1975). Another approach has been to explore inspirational motivation as the result of using concept-based language such as alternative, commitment, work, and so on ( Lord and Emrich, 2000). In sum, understanding the idea of inspirational motivation as a unique construct or establishing its uni-dimensionality has been difficult. Despite this challenge, efforts to separate inspiration and motivation and study inspiration as a separate construct has not been attempted in leadership literature and it is the work of Thrash and Elliot (2003) that has become foundational to explore inspiration as a construct.

Broad audience literature uses the word inspiration rather than inspirational motivation. Anecdotal evidence of the role of inspiration in the performance of an individual is rich in the business world; for instance, job interviewers frequently ask the interviewee ‘what inspires you?’, and citations for awards suggest the inspirational action of an individual as the rationale for the award. Inspiration in its many manifestations is not limited to the domain of personal life (e.g., inspiration by a religious or political leader), but transcends to organizational settings ( Avramenko, 2014), which is perhaps the reason why business leaders such as Steve Jobs had been given the tag of inspiring leaders.

From the above discourse, it emerges that inspirational motivation is not a construct good enough to replace inspiration. Therefore, it is imperative to have a unified understanding of the concept of inspiration differentiated from inspirational motivation. This research addresses the above-mentioned gap by providing a unifying structure for theory building and research on inspiration and provides an overview of emergent constructs and approaches in inspiration research. The incremental contribution of this paper is that 1) the study brings a better understanding of inspiration through a systemic literature review (SLR), and 2) aids to decouple inspiration and motivation and makes a case to augment study inspiration as a separate construct. The authors reviewed extant literature on inspiration between 2003 and 2022 and enhanced the focus of research on inspiration in the organizational context.

## Methods

This systematic review was conducted using the PRISMA (Preferred Reporting Items for Systematic reviews and MetaAnalyses) methodology, which is a recognised approach from Liberati *et al.* (2017). The review was conducted on papers published from 2003 to 2022 to ensure that we had compiled a list of relevant papers that was as complete as possible. The PRISMA Checklist and Abstract Checklist have been reported by the authors under the reporting guidelines ( Vagisha, 2023).

### Eligibility criteria

For the selection of the documents for the systematic review, the following inclusion criteria were determined:

• Regarding the language, only studies that were in English were accepted. Studies that were in other languages were excluded, even if the abstract was in English.
• Regarding the format, only articles that came from specialized management journals were accepted. Articles published on nonspecialized web pages, blogs, or digital newspapers, as well as books, book chapters, or doctoral theses, among others, were excluded.
• Regarding the type of research, only empirical studies were accepted. Theoretical and reflective articles were excluded.
• To adequately address the research questions asked, experimental studies that did not specify their sample were also excluded.

### Search strategy

To compile the studies analyzed in the systematic review, a search protocol was carried out. The databases used to carry out the intended bibliographic review were Ebscohost, ProQuest, Science Direct, and Scopus and the search was carried out from 15 June to 31 August 2022 on all databases. The analyzed period corresponded to the last nineteen years (2003-2022) and the search string used was: ‘inspiration’ OR ‘inspirational leadership’, OR ‘inspirational communication’ OR ‘inspire’ OR ‘inspirational’ OR ‘inspiring’, OR ‘inspired’ in English. The search was run by title, abstract, and keywords. The chosen descriptors were selected to characterize the research in the field of inspiration.

Based on the previously defined parameters, various documents were selected and exported to a specific folder in the Endnote software, which allowed duplicate documents to be identified based on the digital object identifier (DOI) and the bibliographic reference of the source. Subsequently, the articles whose title, keywords, abstract, and content were not directly related to the research questions or did not meet all the inclusion criteria previously described were refined.

### Study selection

For every retrieved paper, the title, abstract, and keywords were confronted with the eligibility criteria. This phase evolved the reading of each abstract of the retrieved papers in an unblinded standardized manner by two researchers, independently. In cases where there was no consensus, the articles were retained for more careful analysis in the phase of full-text analysis.

### Data collection process

We developed a data extraction sheet for the data collection process, considering the Systematic Review goals to ensure that all the pertinent data was retrieved. We The considered variables that started to be collected were the Article Data: type of publication, year, keywords and study variables. The study variables contributed significantly to identifying the emergent themes in the literature. To specifically understand the advancement in the methodological approaches in the literature, we also recorded instruments used, sample characteristics, and selection in the retrieved papers.

### Assessment of risk of bias

The assessment of Risk of Bias of the retrieved papers is done using ROBIS Tool. It assesses both the risk of bias in a review and (where appropriate) the relevance of a review to the research question at hand. Specifically, it addresses 1) the degree to which the review methods minimised the risk of bias in the summary estimates and review conclusions, and 2) the extent to which the research question addressed by the review matches the research question being addressed by its user (e.g. an overview author or guideline developer). Bias occurs if systematic flaws or limitations in the design, conduct or analysis of a review distort the results. Evidence from a review may have limited relevance if the review question did not match the overview/guidelines question.

## Results

The initial data search yielded a total of 224 articles. Based on the inclusion criteria and screening process, 26 articles were included in the review. Table 1 provides the overview of the journals and articles.

**Table 1.** List of journals and frequency of articles.

Journals	Year of Publication																				
	2003	2004	2005	2006	2007	2008	2009	2010	2011	2012	2013	2014	2015	2016	2017	2018	2019	2020	2021	2022	Total
Academy of Management Perspectives									1												1
British Journal of Social Psychology			1																		1
Journal of Business Venturin					1																1
Leadership and Organization Development					1				1				1								3
Organisational Behavior and Human Decision Processes									1												1
Organisation Science							1														1
Journal of Personality and Social Psychology	1	1						2													4
Personality and Social Psychological Bulletin										1											1
Personnel Psychology	1												1								2
Psychology of Women Quarterly									1												1
Social Psychological and Personality Science											1		1								2
The Leadership Quarterly		1			1						1		1								4
Employee Relations															1						1
Human Resource Management																	1				1
Journal of Business Research																		1			1
Journal of Social Psychology																				1	1
Total	2	2	1	0	3	0	1	2	4	1	2	0	4	0	1	0	1	1	0	1	26

### Conceptualization of inspiration

Although inspiration appears to be a conversant term, it has received little sustained attention in academia. It has been conceptualized narrowly within certain content domains (e.g., religious, creative, and interpersonal) or theoretical frameworks, as the authors indicated earlier in this study. The concept of inspiration has been employed in many disciplines including psychology ( Thrash and Elliot, 2003), anthropology ( Leavitt, 1997), theology ( Aquinas, 1950; Canale,1994a, 1994b), education ( Tjas *et al.,* 1996), art and literature ( Bowra, 1955; Harvey, 1999), engineering ( Beer *et al.,* 1997), management ( Bass and Avolio, 1994; Dess and Picken, 2000), and communication ( Frese *et al.,* 2003; Rafferty and Griffin, 2004).

The year 2003 saw a seminal contribution in empirical research on inspiration. The significant section of empirical works on inspiration is ruled by two scholastic spaces: psychology and leadership. The three essential qualities of the state of inspiration are evocation, transcendence, and motivation, according to the tripartite conceptualization ( Thrash and Elliot, 2003). Evocation suggests that inspiration is aroused by an external source and is involuntary. Inspiration involves the energization and direction of behavior. Transcendence suggests that inspiration attunes one toward something that is beyond one’s habitual limits enabling one to see better possibilities ( Thrash and Elliot, 2003).

Although the tripartite conceptualization provides a useful characterization of the state of inspiration, important questions remain regarding the processes that give rise to these characteristics. Thrash and Elliot (2004) proposed that inspiration is a hybrid construct that emerges from the juxtaposition of two component processes. The first component involves an appreciation of and accommodation or adaptation to an evocative object which is referred to as ‘inspired by’. The second component involves a drive to imbibe the qualities demonstrated in the evocative object/person (referred to as ‘inspired to’).

The broad concept of inspiration discussed earlier can be directly extended to the sphere of creative action ( Thrash and Elliot, 2003). Thrash and Elliot’s (2003) research related general trait inspiration to creativity. Even when creative self-concept was controlled, trait inspiration still predicted mean levels of self-reported creativity in daily life. Additionally, among a sample of inventors, the frequency of inspiration predicted the number of U.S. patents received. This latter finding is crucial since patents are a concrete measure of creative achievement ( Thrash and Elliot, 2004). Thrash and Elliot (2003) reported inspiration could be an antecedent of creativity and that people’s creativity tends to depend on inspiration. Self-reported creativity and general state inspiration fluctuate within individuals, over time. This suggests that people could be more creative on days when they feel more inspired. In other words, inspiration could be studied as an antecedent of creativity.

### Emergent themes

*Trait assessment scale of inspiration*

Inspiration is both a state and a personality attribute ( Thrash and Elliot, 2004). These are thought to vary between and among individuals. Thrash and Elliot (2004) developed the inspiration scale (IS) as a trait assessment of inspiration. Even if the term ‘trait’ has many meanings, it relates to personal differences in the tendency to experience the condition of inspiration. The IS has two internally consistent four-item subscales: inspiration frequency and intensity. Both subscales have Cronbach’s coefficients of 0.90 or above, indicating that they are internally consistent. The IS demonstrates measurement invariance across time (two months) and demographics (patent holders and university grads), implying that the basic latent components have similar meaning across time and groups. Both subscales exhibit strong test-retest reliabilities of two months (r = 0.77). In a nutshell, the IS has outstanding psychometric properties. The intensity subscale was created as a status indicator ( Thrash and Elliot, 2004; Thrash, Elliot *et al.,* 2010).

*Wellbeing*

Thrash, Elliot *et al.* (2010) posited that inspiration promotes different forms of wellbeing, including ‘hedonic wellbeing’ (e.g., activated positive affect), which is ‘pleasure-oriented’, and ‘eudemonic wellbeing’ (e.g., self-actualization), which is ‘growth-oriented’. The component processes of being inspired by and to were theorized to foster gratitude and purpose in life, respectively, and gratitude and purpose were posited to mediate effects on a variety of wellbeing. Inspiration consistently resulted in enhancements in activated “positive affect, life satisfaction, vitality, and self-actualization” throughout a series of research designed for causal inference, including an experiment, two cross-lagged longitudinal studies, and a diary study ( Thrash *et al.,* 2010).

In a more recent study, Milyavskaya *et al.* (2012) found that inspiration predicts achievements of individual goals. They also highlighted that goal progress may act as an additional mediator of the impact of inspiration on wellbeing. According to Straume and Vittersø (2012), inspiration is a sign of eudemonic wellbeing in and of itself.

*Role models*

Lockwood *et al.* (2002) investigated inspiration in terms of good and negative role models using three experiments with three different populations. They used regulatory focus questionnaires, role model adjustment ratings, and motivation ratings. Hoyt and Simon (2011) looked at whether upward social comparisons to high-level female leaders had a detrimental impact on women’s self-perception and aspiration and discovered those female role models who contradicted the negative stereotype had a much more positive impact. People are motivated to compare themselves to superior role models to seek inspiration and hope ( Hoyt and Simon, 2011). Role models at any level can be inspiring to the extent that individuals identify with them, consider their success as achievable, and “successfully disconfirm, at an explicit level, gender-stereotypical beliefs” ( Hoyt and Simon, 2011).

*Leadership*

Molenberghs *et al.* (2015) and Waldman *et al.* (2011) studied the role of neuroscience in inspirational leadership and provided information on underlying brain processes associated with inspirational communication. Searle and Hanrahan (2011) used a phenomenological approach to study the lived and personal experiences of leaders, looking at inspiration as a psychological concept in the context of leadership. According to Rafferty and Griffin (2004), inspiring communication is one of the subdimensions of transformational leadership, in which leaders use inspirational appeals and emotive rhetoric to elicit followers’ motivation to move beyond self-interest for the good of the team Boies *et al.* (2015). Frese *et al.* (2003) presented and evaluated a management action training program that included inspirational vision communication as part of charismatic leadership training. They observed being inspirational as one aspect of charismatic leadership. Salas-Vallina and Fernandez (2017) investigated the relationship between inspirational leadership, participatory decision-making, and happiness at work. They found that participatory decision-making acts as a complete intermediary in the relation between inspired leadership and happiness at work. According to Mitchell and Boyle (2019), followers’ good moods tend to mediate the path between inspirational leadership and innovation, which could explain its various benefits. Inspiring leaders boost the team’s morale, which leads to more creative and flexible thinking. Mitchell and Boyle (2019) found that professional salience acts as a critical boundary condition in this relationship, with inspirational leaders increasing multidisciplinary team innovation through positive mood when a profession is salient. Salas-Vallina *et al.* (2020) looked at how inspirational leaders influence followers’ personality attributes and, as a result, their job happiness. Followers are motivated to actively learn by the intellectual stimulation provided by charismatic leaders. Leaders that instill a sense of responsibility and authority in their followers improve their employees’ quality of life ( Salas-Vallina *et al.,* 2020).

*Social comparison*

Burleson *et al.* (2005) and Lockwood *et al.* (2012) looked at the role of social comparison in understanding changes in a teen’s creative self-concept when looking at inspiration. They assessed unfavorable comparisons that created a sense of inferiority, as well as favorable parallels that instilled a sense of inspiration. They connected comparisons of inferiority to negative changes in self-esteem. They linked improvements in self-esteem to inspirational comparisons made during the training. Upward comparisons inspire people to make the transition to a new cultural setting, according to Lockwood *et al.* (2012).

*Social learning*

Xia and Li (2022) introduced a new pasture of the role of inspiration in facilitating people’s social learning and communication across settings. To advance the understanding of how people share what they know, they examined the association between the inspiring level of a given message and its likelihood of being shared. The researchers reported a positive association between them.

### Advancement in methodological approaches

*Instruments used*

Thrash and Elliot (2003) developed the IS for their study. Subsequently, other authors ( Thrash and Elliot, 2004; Thrash, Elliot *et al.,* 2010; Thrash, Maruskin *et al.,* 2010) used the IS for the research. Most authors ( Hoyt, 2013; Hoyt and Simon, 2011; Lockwood *et al.,* 2002, 2012; Parr *et al.,* 2013; Souitaris *et al.,* 2007; Stephan *et al.,* 2015; Thrash *et al.,* 2010; Van Kleef *et al.,* 2015) used self-reported five-point scale to measure inspiration. Molenberghs *et al.* (2015) used the 204-item battery for visionary leadership ( Kahan, 2002). Visionary leadership is transformative and based on the power of inspiration. Sosik and Dinger (2007) used Crowne and Marlowe’s (1960) social desirability scale-personal attributes shortened Reynolds’ (1982). Sosik and Dinger (2007) also used Good and Good’s (1972) 28-item dichotomously score to investigate inspiration through social power motivation. They also used Snyder and Gangestad’s 18-item scale to study the relationship between leaders’ personal attributes, leadership style, and inspirational vision themes. Table 2 offers an overview of the measurements used.

**Table 2.** Instruments used for measurement.

Instruments	N *	% of Codable Studies
Multifactor Leadership Questionnaire	5	22.73
Five-point scale	3	13.64
Leach *et al.,*’s (2003) Social Comparison Questionnaire	1	4.55
Seven-point scale	4	18.18
11-point scale	1	4.55
Web surveys	1	4.55
204 item battery for visionary leadership and pre-task information sheet	1	4.55
House’s (1998) three items to measure inspirational communication	1	4.55
In-depth interviews	1	4.55
Social Desirability Scale-personal attributes	1	4.55
Good and Good’s (1972) 28-item dichotomously score	1	4.55
IS ( Thrash and Elliot, 2003)	4	18.18
Self-report measure of social power motivation	1	4.55
Snyder and Gangestad’s (1986) 18-item scale	1	4.55

Note: All the articles were codable based on the instruments used for the measurements.

* Overlapping of the codable studies is attributed to the use of multiple instruments in the same study.

*Sample characteristics and selection*

The authors reviewed the characteristics of the sample to determine the commonalities and differences in the target respondents and the extent to which these were generalisable to populations of interest. They reviewed 26 papers, out of which 18 papers comprehensively discussed sample characteristics. Seven (of 26) papers did not indicate the gender of the respondents and two (out of 26) did not indicate the occupation of the sample. Table 3 provides an overview of sample criteria and characteristics.

**Table 3.** Sample criteria and characteristics.

Category	Codable Studies	N	% of Codable Studies
**A. Gender of sample**			
Female-only	18	3	16.67
Male-only		0	0
Mainly female		10	55.55
Mainly male		4	22.22
Gender-balanced		1	5.56
**B. Occupation**			
Students	23	12	52.17
High school artists		1	4.13
Undergraduates		11	47.82
Midlevel managers		1	4.13
Fundraisers		1	4.13
Customer service employees		1	4.13
Marketing managers		1	4.13
Public sector employees		1	4.13
Mixed		2	8.69
Focal leaders		1	4.13
Medical specialists		1	4.13
Healthcare teams		1	4.13
Frontline banking employees		1	4.13
**C. Geographic region**			
United States *	14	7	50
Europe		2	14.28
Canada		2	14.28
India		1	7.14
Australia		1	7.14
United Kingdom		1	7.14

* Research setting: U.S. East Coast, Southeast U.S. or Western U.S.

Gender of the sample. The authors coded the gender information in the studies as female-only, male-only, mainly female, mainly male, and gender-balanced. Of the 26 studies, 18 studies were codable and the remaining five studies did not indicate any information about the gender of the sample. Three studies used only female samples; 10 studies used mainly females as the sample; four studies used mainly males, and only one study was gender balanced.

Occupation of the sample. 23 studies were codable as per the information indicated regarding the occupation. 12 (out of 23) studies where the occupation was coded indicated students were predominant in the sample selection. The researchers sub-coded the category of students as high school students and undergraduates wherein the latter was predominant. The sample of undergraduates included students of liberal arts, introductory psychology, personality psychology, science, and engineering. Two studies were based on a mixed sample. Six studies were coded as midlevel managers, fundraisers, customer service employees, public sector employees, focal leaders, and mixed. The sampling frames of the two studies were made up of allergy specialists from Spanish public hospitals and multidisciplinary healthcare teams based in the United Kingdom. Frontline banking personnel at Italian and Spanish banks were considered in one study.

Geographic region. 14 out of the 26 studies were codable for geographic regions. Seven (out of 14) were conducted in the United States, two studies each in Canada, Europe, India, and Australia, and one study in the United Kingdom.

The findings of the study are:

• 26 out of 224 articles were identified for the final review. The excluded articles did not meet the either one or all of the inclusion criteria.
• Inspiration has been well understood intuitively for centuries but is nascent as an academic construct ( Thrash and Elliot, 2004).
• Inspiration as a term has been used in leadership literature widely to depict a distinct type of leadership that is superior to other forms, but the construct of inspiration itself has received little attention in conceptual or empirical research till this century ( Thrash and Elliot, 2003).
• The term has been used to denote higher order motivation and is distinct enough from motivation to demand theory building (Locke, 1982).
• The term has been used in theology ( Canale, 1994a, 1994b), literature ( Bowra, 1955; Harvey, 1999), art ( Bowra, 1955; Harvey, 1999), management ( Bass and Avolio, 1994; Dess and Picken, 2000), and several other areas ( Beer, *et al.,* 1997; Hart, 1998; Kris, 1952; Lockwood and Kunda, 1997), which suggests that there is a need to have research that led to a general theory of inspiration.
• Inspiration, as a construct, is recent ( Thrash and Elliot, 2003) and has been emerging through a series of studies between 2003 and 2022.
• Robust instruments have been developed to measure inspiration and this is likely to make an impetus in the research on inspiration, but more research in scale building is required ( Thrash and Elliot, 2003; Hoyt, 2013; Hoyt and Simon, 2011; Lockwood *et al.,* 2002; Lockwood *et al.,* 2012; Parr *et al.,* 2013; Souitaris *et al.,* 2007; Stephan *et al.,* 2015; Thrash *et al.,* 2010; Van Kleef *et al.,* 2015; Molenberghs *et al.,* 2015; Crowne and Marlowe, 1960; Good and Good, 1972; Snyder and Gangestad, 1986).
• There have been several avenues for studying inspiration: Antecedents of creativity, a component of charismatic leadership ( Frese *et al.,* 2003), dimension of transformational leadership ( Rafferty and Griffin, 2004), its role in social learning ( Xia and Li, 2022), social comparison ( Burleson *et al.,* 2005; Lockwood *et al.,* 2012), well-being (Thrash *et al.,* 2010), and role model ( Hoyt and Simon, 2011; Lockwood *et al.,* 2002).
• Empirical studies on inspiration are emerging and are poised to fill a key knowledge gap on how people respond to inspiration and go on to achieve exceptional performance.
• A detailed search of the literature revealed that there is a vast knowledge gap awaiting empirical research which can have a far-reaching impact on society and management; for instance, the impact of inspiration on performance and the role of moderators such as spirituality, visioning capability, gender variation, and linguistic proficiency.

## Discussion

Inspiration has been well understood intuitively for centuries but is nascent as an academic construct ( Thrash and Elliot, 2004). Inspiration as a term has been used in leadership literature widely to depict a distinct type of leadership that is superior to other forms, but the construct of inspiration itself has received little attention in conceptual or empirical research till this century ( Thrash and Elliot, 2003). The term has been used to denote higher order motivation and is distinct enough from motivation to demand theory building (Locke, 1982). The term has been used in theology ( Canale, 1994a, 1994b), literature ( Bowra, 1955; Harvey, 1999), art ( Bowra, 1955; Harvey, 1999), management ( Bass and Avolio, 1994; Dess and Picken, 2000), and several other areas (( Beer, *et al.,* 1997; Hart, 1998; Kris, 1952; Lockwood and Kunda, 1997), which suggests that there is a need to have research that led to a general theory of inspiration. Inspiration, as a construct, is recent ( Thrash and Elliot, 2003) and has been emerging through a series of studies between 2003 and 2022. Robust instruments have been developed to measure inspiration and this is likely to make an impetus in the research on inspiration, but more research in scale building is required ( Thrash and Elliot, 2003; Hoyt, 2013; Hoyt and Simon, 2011; Lockwood *et al.,* 2002; Lockwood *et al.,* 2012; Parr *et al.,* 2013; Souitaris *et al.,* 2007; Stephan *et al.,* 2015; Thrash *et al.,* 2010; Van Kleef *et al.,* 2015; Molenberghs *et al.,* 2015; Crowne and Marlowe, 1960; Good and Good, 1972; Snyder and Gangestad, 1986).

There have been several avenues for studying inspiration: Antecedents of creativity, a component of charismatic leadership ( Frese *et al.,* 2003), dimension of transformational leadership ( Rafferty and Griffin, 2004), its role in social learning ( Xia and Li, 2022), social comparison ( Burleson *et al.,* 2005; Lockwood *et al.,* 2012), well-being (Thrash *et al.,* 2010), and role model ( Hoyt and Simon, 2011; Lockwood *et al.,* 2002). Empirical studies on inspiration are emerging and are poised to fill a key knowledge gap on how people respond to inspiration and go on to achieve exceptional performance. A detailed search of the literature revealed that there is a vast knowledge gap awaiting empirical research which can have a far-reaching impact on society and management; for instance, the impact of inspiration on performance and the role of moderators such as spirituality, visioning capability, gender variation, and linguistic proficiency.

Despite being a concept of long-established interest in varied disciplines, inspiration has gained little empirical attention. There is an absence of systematic analysis of conceptualization as well as of various approaches to research methods across inspiration literature. It is evident from the review that research on inspiration is flourishing but is fragmented. It is indicative that inspiration scholarship needs to bring definitional and conceptual concurrence, so that the foundation for research and theory building becomes robust.

This paper brings together the emergent constructs of inspiration from the literature and methodological approaches of the studies *.* There is a scope for a different selection criterion for the target respondents, as the present study indicates that most of the research targets undergraduates, majorly. It seems appropriate to include working professionals and corporate and social leaders. The findings have substantial implications for understanding and taking forward the contemporary inspiration literature by laying down a comprehensive overview of the extant scholarship of inspiration and identifying various gaps.

It is observed that a significant number of articles revolves around the theme of leadership, which could be attributed to use of inspiration by the leaders to achieve exceptional outcomes. This underscores the importance of knowing about inspiration so that everyone could use the tools of inspiration to achieve exceptional outcomes.

Though this study includes all factors utilised in the study of inspiration, it is limited to journal articles and does not include published books, conference papers or other referred or non-refereed sources. The study had an “empirical articles” focus, and the inclusion of non-empirical studies could enhance the knowledge in this area.

The current literature on inspiration stops with a few empirical studies and tools, and this study recommends that research on inspiration focuses to crystallize inspiration as a construct, identify various dimensions of inspiration, and then firm up a general theory of inspiration with robust empirical evidence. There is a need to expand the scope of the IS by developing and trying out newer competing scales.

## Data Availability

DANS EASY: Mapping inspiration in human resources: A systematic review of themes and approaches. https://doi.org/10.17026/dans-zr5-73km ( MAHE, 2022). The project contains the following underlying data:
• Data.pdf (Inspiration SLR Data in Tables 1, 2, and 3) Data are available under the terms of the Creative Commons Zero “No rights reserved” data waiver (CC0 1.0 Public domain dedication). FIGSHARE: PRISMA and PRISMA abstracts checklists for Mapping inspiration in human resources: A systematic review of themes and approaches’. https://doi.org/10.6084/m9.figshare.21947957. Data are available under the terms of the Creative Commons Zero “No rights reserved” data waiver (CC0 1.0 Public domain dedication).
