# Supplementary material for: Mapping inspiration in human resources:  A systematic review of themes and approaches
Source: F1000Res. 2023 Jun 16;12:694. [Version 1] doi: 10.12688/f1000research.128853.1 (PMC10448870; doi:10.12688/f1000research.128853.1)
Supplement: Peer review history

# Peer review: Reviewer response for version 1

**DOI:** 10.5256/f1000research.141484.r194564

**Published:** 2023-08-23

**Authors:** Idris Idris

**Affiliations:** 1 Universitas Negeri Malang, Malang, East Java, Indonesia

**Introduction:**

 An overview of leadership literature, the sentence “none of them have explained what inspiration is” should be compared to other previous literature, at least what they were conducted in terms of inspiration that emerged in their studies. In addition, the research question in a systematic literature review is compulsory.

**Method:**

 The research procedure was presented in detail. But, this manuscript failed to follow the one crucial step in SLR, formulating the research question. This step will specify what type of studies can best address that question and set out criteria for including such studies in the review (inclusion criteria or eligibility criteria). Any research question has ideological and theoretical assumptions around the meanings and processes it is focused on. A systematic review should either specify definitions and boundaries around these elements at the outset or be clear about which elements are undefined, including in the search string used and broken down from the research questions. 

 Another issue is about data analysis used in the review. It is crucial for this study to enhance and formulate the data extraction and analysis and draw the findings of the study. 

**Results:**

 In the results, the authors should present the PRISMA figure mentioned in the study, such as how many articles in total were obtained based on the search string and excluded based on the eligibility criteria.

**Discussion:**

 What is presented in the discussion section is better to follow the research goals or research questions.

**Conclusion:**

 No conclusion is presented in the manuscript.

Are the rationale for, and objectives of, the Systematic Review clearly stated?

Yes

Is the statistical analysis and its interpretation appropriate?

Partly

Are sufficient details of the methods and analysis provided to allow replication by others?

Partly

Are the conclusions drawn adequately supported by the results presented in the review?

No

Reviewer Expertise:

My research interest includes but is not limited to Organizational behavior, leadership, and tourism and hospitality.

I confirm that I have read this submission and believe that I have an appropriate level of expertise to confirm that it is of an acceptable scientific standard, however I have significant reservations, as outlined above.

# Peer review: Reviewer response for version 1

**DOI:** 10.5256/f1000research.141484.r194563

**Published:** 2023-08-18

**Authors:** Hussam Ali Mhaibes

**Affiliations:** 1 University of Baghdad, Baghdad, Iraq

I commend the researchers for their diligent work, as their study delves into a significant subject matter in an intriguing manner, offering a unique scientific contribution to an area already explored by scholars.

 While the research is commendable, I have a few observations that, if addressed, could further enhance the quality of the study:

1. The research objective, although present, could benefit from greater clarity in its expression. It appears that the researchers may have slightly underestimated the significance of their research objective, warranting a more comprehensive portrayal.
2. The mention of the sources originating from Ebscohost, ProQuest, Direct Science, and Scopus might not be necessary. The research itself is lucid, and there's no need to reiterate that the selected sources were of quality. Rather, it would be sufficient to state that the research selected for review was well-chosen.
3. Given that a substantial number of sources cited by the researchers pertain to "Inspirational Leadership," it might be advisable to consider incorporating this term into the research title. This approach could effectively encapsulate the research's essence and its focus on this particular area.
4. The introduction contains solid theoretical groundwork, yet it lacks certain fundamental elements typically expected in an introduction. Specifically, the final paragraph of the introduction seems to leave room for further elaboration and development.

In conclusion, the results and conclusions of the study are commendable and have been thoughtfully discussed. Moreover, the selection and formatting of references have been executed with precision.

 Thank you for considering these observations. I believe that addressing these points could contribute to refining the research and enhancing its impact. I appreciate the opportunity to engage with this scholarly work and contribute to its improvement.

Are the rationale for, and objectives of, the Systematic Review clearly stated?

Partly

Is the statistical analysis and its interpretation appropriate?

Yes

Are sufficient details of the methods and analysis provided to allow replication by others?

Yes

Are the conclusions drawn adequately supported by the results presented in the review?

Yes

Reviewer Expertise:

General specialization in public administrationSpecialization in strategic management and organization theory

I confirm that I have read this submission and believe that I have an appropriate level of expertise to confirm that it is of an acceptable scientific standard.

# Peer review: Reviewer response for version 1

**DOI:** 10.5256/f1000research.141484.r181652

**Published:** 2023-07-19

**Authors:** Ashish Bollimbala

**Affiliations:** 1 School of Management, Indian Institute of Technology Mandi, Mandi, Himachal Pradesh, India

The study fills a major gap in inspirational literature which is sparse and has the potential to kick start deliberate and scientific studies in the concept of inspiration which is highly valued, at least in general literature. The work has the potential to make research in inspiration valuable and so, it is an important contribution to the knowledge on inspiration. However, the authors could address the following issues to enhance the value of this paper.

1. Abstract – The authors are advised to revise the abstract to revise the findings/conclusions so that a reader can get a clearer picture of the value of the study. This is important to attract readership to the article and consequently to the journal. For instance, the findings could highlight the themes that have emerged from the analysis of literature. 
2. Key Words – More attention could be given to the key words which enables finding the article against search. For example, a key word such a ‘review’ may be too general to be of much use.
3. Rather than state that various sites have been searched, it is preferable to be more specific and clarify the number of electronic databases used.
4. It is important to distinguish between inspiration and motivation. Mere statements that inspirational motivation is different from inspiration is not sufficient to justify that inspiration and motivation are or at the least possibly different constructs. This assertion with literature support is crucial for progression of research in inspiration using it as a distinct construct different from motivation. This is an important aspect the authors should address.
5. While the authors state “From the above discourse, it emerges that inspirational motivation is not a construct good enough to replace inspiration”, it is recommended that some examples of broad audience literature with reference is used. Though this might be intuitive or even correct, referencing is the key to a good research article.
6. The authors state “The researchers obtained the abstract from the databases of EbscoHost or ProQuest or Science Direct”. Is it that they procured it using all these databases? The word ‘or’ could be confusing. If they authors have used all of them, the conjunction ‘and’ would be more appropriate than ‘or’.
7. The period of inclusion is from 2003 to 2022. While 2022 needs no justification, reason for using 2003 as the start point for the database search needs some elaboration. At the moment it looks a little arbitrary.
8. Para 3.1, Conceptualization of Inspiration should be rewritten to improve the flow. Thereafter, the emergent themes included in 3.2 are well addressed.
9. From a plain reading of Para 3.3, and the articles in general, it appears that Thrash and Elliot makes a significant contribution and there is an important landmark in inspiration research. It will help the reader if the authors elucidate this instrument a little more and even include some of the qualitative strengths of the instrument such as reliability and validity so that it helps future research.
10. Though minor, changing the language such as ‘gender of the sample’ or ‘occupation of the sample’ can have a salutary effect on the quality of the paper.
11. The findings are comprehensive and well addressed.
12. The last para addresses future areas of study. Since the authors have done so much study and are suggesting the importance of addressing the concept more empirically, even though such suggestion may be tacit, could the authors consider suggesting the general direction of empirical studies with some examples so that research in inspiration can be invigorated?

Are the rationale for, and objectives of, the Systematic Review clearly stated?

Yes

Is the statistical analysis and its interpretation appropriate?

Yes

Are sufficient details of the methods and analysis provided to allow replication by others?

Partly

Are the conclusions drawn adequately supported by the results presented in the review?

Partly

Reviewer Expertise:

Behavioral science.

I confirm that I have read this submission and believe that I have an appropriate level of expertise to confirm that it is of an acceptable scientific standard, however I have significant reservations, as outlined above.
